# Natural convection flow of a second grade fluid in an infinite vertical cylinder

**DOI:** 10.1038/s41598-020-64533-z

**Published:** 2020-05-20

**Authors:** Maria Javaid, M. Imran, M. A. Imran, I. Khan, K. S. Nisar

**Affiliations:** 1 Department of Mathematics, Government College University, Faisalabad, Pakistan; 2 Department of Mathematics, University of Management and Technology Lahore, Lahore, 54770, Pakistan; 3 Faculty of Mathematics and Statistics, Ton Duc Thang University, Ho Chi Minh City, 72915 Viet Nam; 4 Department of Mathematics, College of Arts and Sciences, Prince Sattam bin Abdulaziz University, Wadi Aldawaser, 11991 Saudi Arabia

**Keywords:** Applied mathematics, Physics

## Abstract

In current study natural convection flow of second grade fluid in an oscillating infinite vertical cylinder is investigated. The dimensionless governing equations for temperature and velocity are obtained by introducing the non-dimensional variables. Exact solutions for temperature and velocity field are computed by means of integral transformation. Solutions for cosine and sine oscillations of velocity field are introduced in the form of transient and post-transient arrangements. A special case for Newtonian fluid is obtained from general results and transients solutions are computed in terms of tables. In the end, the impact of dimensionless numbers (Grashof and Prandtl numbers) at different values of time is presented in graphical form and found that velocity for Newtonian fluid has greater values than the second grade fluid. Furthermore, there are some comparisons of calculated solutions with existing solutions in literature.

## Introduction

Due to complicated relation between stress and strain in non-Newtonian fluids and their technological application, their study in fluid dynamics is more valuable^1^ than Newtonian fluids. Viscous fluids flow has attracted the attention of scientists and engineers because of its important applications notably in the flow of oil through porous rocks, the extraction of energy from geothermal regions, the filtration of solids from liquids and drug penetration through human skin. Second grade fluid is a subclass of non-Newtonian fluid in which velocity field has up to two derivatives in stress strain tensor relationship where as in Newtonian fluid it has derivatives up to first order. Flow of second grade fluid gains attention of the researchers in many boundary layer flows and have been successfully studied in various kinds of flows. Study of heat transfer in non-Newtonian fluids is much interesting for researchers now-a-days. The influences of temperature dependent viscosity on second grade fluid causes changes in the properties of the fluid. For the case of gases, the viscosity of the gas increases as heat given to gas while for liquid it becomes thin as temperature increases. As a result many scientist devoted to study the effects of variable viscosity models. Fetecau^2–4^ worked on the uniqueness of some helical flows of a second grade and Oldroyd-B fluids in cylindrical domains, they studied the fluid motion by applying time dependent stress on the boundary and studied the effects of physical parameters on the fluid motion. Further Jamil and Khan^5^ used the same technique for Burger’s fluid and obtain the result for velocity and shear stress. Keeping in view the importance of fluid flow in cylindrical domain researchers studied fluid motion in cylinders by considering different fluids and boundary conditions. Barnes *et al*.^6,7^ analyzed the polymer flow in circular cylinder by considering pulsatile APG, Davies *et al*.^8^ and Phan-Thien^9^ worked on the same problem for White-Metzner fluid. Jamil *et al*.^10^ obtained exact solutions for the motion of a fractionalized second grade fluid due to longitudinal and torsional oscillations of an infinite circular cylinder are determined by means of Laplace and finite Hankel transforms. Fetecau *et al*.^11^ obtained the solutions for the oscillating motion of a generalized Burgers fluid due to longitudinal oscillations of an infinite circular cylinder, as well as those corresponding to an oscillating pressure gradient, are established as Fourier–Bessel series in terms of some suitable eigenfunctions. The propagation of a heat wave in an incompressible second grade fluid within the context of a potential vortex was studied by Fetecau *et al*.^12^. Flow induced by non-coaxial rotation of a porous disk executing non-torsional oscillations of a second grade fluid was studied by Hayat *et al*.^13^. The fractional calculus approach in the constitutive relationship model of second grae fluid was introduced and the flow characteristics of the viscoelastic fluid in double cylinder rheometer were studied by Huang *et al*.^14^.

In fluid motion free convection is induced by buoyancy forces. These forces may arise due to density gradients and body forces. In free convection the Grashof number plays the same role as in forced convection the Reynolds number. The Grashof number is actually the ratio of buoyancy forces to viscous forces. Heat transfer due to convection has several industrial and technological applications. Their examples may be found in wire and fiber coating, manufacturing plastic films, artificial fibers, polymer sheets, chemical processing equipment and in the design of various heat exchangers. Mixed convection heat transfer in a horizontal channel filled with nanofluids studied by Fan *et al*.^15^. Gul *et al*.^16^ studied heat transfer in MHD mixed convection flow of a ferrofluid along a vertical channel and found that temperature and velocity of ferrofluids depend strongly on viscosity and thermal conductivity together with magnetic field. Gul *et al*.^17^ studied energy transfer in mixed convection MHD flow of nanofluid containing different shapes of nanoparticles in a channel filled with saturated porous medium. They concluded that viscosity and thermal conductivity are the most prominent parameters responsible for different results of velocity and temperature. Due to higher viscosity and thermal conductivity, C_2_H_6_O_2_ is regarded as better convectional base fluid compared to H_2_O. Chamkha^18^ studied non-similar solutions for mixed convective boundary layer flow a non-Newtonian fluid over a wedge embedded on a porous medium filled with a nanofluid. Sheikhzadeh *et al*.^19^ performed parametric study on MHD mixed convection of Cu-water nanofluid in a two-sided lid-driven porous cavity with a partial slip. Prasad *et al*.^20^ studied a convection flow over a permeable non-isothermal wedge and reveal many interesting behaviors and study of the flow and heat transfer characteristics over the permeable wedge. Hasnain *et al*.^21^ studied the effects of porosity and convection on MHD two phase fluid flow in an inclined channel. Straub *et al*.^22^ proposed a simple model that allow estimating the achievable drag reduction rates in duct flows as a function of the width of the duct and the spanwise extent of the controlled region and showed that this spanwise limitation of the oscillating region strongly diminishes the drag reduction potential of the control technique. Straub *et al*.^23^ studied the effect of selected thermal boundary conditions on a fully developed turbulent pipe flow. Younghae *et al*.^24^ studied Navier’s slip condition on time dependent Darcy-Forchheimer nanofluid using spectral relaxation method. A numerical investigation of laminar natural double diffusive convection in an open ended vertical cylindrical annulus with unheated entry and un-heated exit was performed by Sankar^25^. Natural convection flows in a vertical annulus filled with a fluid-saturated porous medium was investigated when the inner wall is subject to discrete heating by Sankar *et al*.^26^.

Shah and Khan studied heat transformation in second grade fluid by using fractional Caputo-Fabrizio derivatives^27^. Fetecau *et al*. observed effects of convection of fractional Oldroyd-B fluid flow with thermal diffusion^28^. Heat transfer in oscillating fractional Maxwell fluid is studied by Khan *et al*.^29^. Free convection flow of a unsteady second grade fluid is calculated by Ali *et al*.^30^. Hayat *et al*.^31^ studied slip flow and heat transfer of a second grade fluid past a stretching sheet through a porous space. Cortell^32^ studied the flow, chemical reaction and mass transfer of a steady laminar boundary layer flow of an electrically conducting fluid of second grade in a porous medium subject to a transverse uniform magnetic field past a semi-infinite impermeable stretching sheet. Ariel^33^ studied axisymmetric flow of a second grade fluid past a stretching sheet by using a perturbation method. Vajravelu *et al*.^34^ studied the flow and heat transfer characteristics in a second grade fluid over a stretching sheet with prescribed surface temperature including the effects of frictional heating, internal heat generation or absorption, and work due to deformation. Natural convection heat transfer for Newtonian fluid in an oscillating vertical cylinder is studied by Khan *et al*.^35^.

The aim of current study is to study the second grade fluid flow and natural convection heat transfer. We are pleased to acknowledge here that there is no work on natural convection heat transfer in cylindrical domains, current work in oscillating cylinder is new one. Integral transform technique is used to find exact solutions for temperature distribution and cosine and sine oscillations of velocity field. Moreover, velocity field is introduced in the form of transient and post-transient arrangements. To check the impact of dimensionless numbers (Grashof and Prandtl numbers) at different values of time, graphical illustrations are used. The transient solutions are also computed in tables. Furthermore, a special case for Newtonian fluid is obtained and a comparison between Newtonian and second grade fluid is depicted by a graph. Moreover, there are comparisons with solutions for second grade fluids obtained in various works by other researchers.

## Problem Formulation and Solution

**Figure 1:**
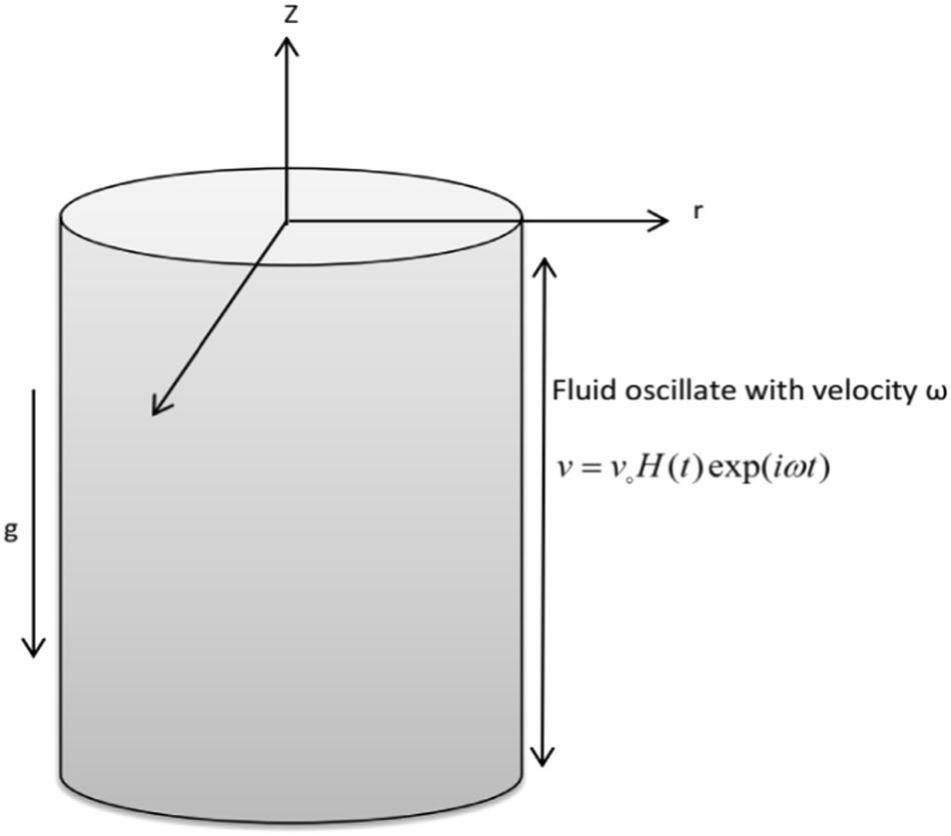
Physical Model of the Problem.

Consider a viscous second grade fluid in a vertical infinite cylinder having radius *R*. The z-axis is considered along the axis of cylinder in vertical upward direction and the radial coordinate *r* is taken normal to it, flow is consider to be unidirectional. Fluid flow is considered along z-direction and there is no fluid motion along *θ* and *r*. At time *t* ≤ 0 cylinder is at rest and both cylinder and fluid are at the same temperature *T*_∞_. After time *t* > 0 cylinder starts to oscillate with frequency *ω* along its axis. At the same time, the cylinder temperature raised to *T*_*w*_ which is thereafter maintained constant as shown in Fig. 1.

We assume that the velocity and temperature are the function of *r* and *t* only. Therefore, velocity and temperature is of the following form^35^,

1 $${\bf{V}}=v(r,t);T=T(r,t).$$

For such a flow, taking the usual Boussinesq approximation, following set of partial differential equations governed the flow^35^,

2 $$\frac{\partial v(r,t)}{\partial t}=\nu \left(\frac{{\partial }^{2}}{\partial {r}^{2}}+\frac{1}{r}\frac{\partial }{\partial r}\right)v(r,t)+\frac{\alpha }{\rho }\frac{\partial }{\partial t}\left(\frac{{\partial }^{2}}{\partial {r}^{2}}+\frac{1}{r}\frac{\partial }{\partial r}\right)v(r,t)+g\beta (T-{T}_{\infty }),$$

3 $$\frac{\partial T(r,t)}{\partial t}=\frac{k}{\rho {c}_{p}}\left(\frac{{\partial }^{2}}{\partial {r}^{2}}+\frac{1}{r}\frac{\partial }{\partial r}\right)T(r,t),$$

where, *v*(*r*, *t*), *v*, *α*, *ρ*, *g*, *β*, *T*, *k* and *c*_*p*_ is velocity, viscosity, material constant, density, gravitational acceleration, volumetric coefficient of thermal expansion, temperature, thermal conductivity and the heat capacity at constant pressure respectively.

The suitable initial and boundary conditions are

4 $$v(r,0)=0,T(r,0)={T}_{\infty },\,r\in [0,R],$$

5 $$v(R,t)={v}_{\circ }H(t)\exp (i\omega t),T(R,t)={T}_{w}\,t > 0,$$

where, *v*_o_ and *H*(*t*) are characteristic velocity and unit step function respectively.

Next we use the non-dimensional variables and functions in order to determine solutions which are independent of the flow geometry,

6 $$\begin{array}{c}{t}^{\bullet }=\frac{{v}_{\circ }{}^{2}t}{\nu },\,{r}^{\bullet }=\frac{{v}_{\circ }r}{\nu },\,{v}^{\bullet }=\frac{v}{{v}_{\circ }},\,\theta =\frac{T-{T}_{\infty }}{{T}_{w}-{T}_{\infty }},\,{\alpha }^{\bullet }=\frac{\alpha {v}_{\circ }{}^{2}}{\mu \nu },\\ {\rm{Gr}}=\frac{\nu g\beta ({T}_{w}-{T}_{\infty })}{{{v}_{\circ }}^{2}},\,{\rm{\Pr }}=\frac{\mu {c}_{p}}{k},\end{array}$$

where, *Gr* and *Pr* are Grashof number and Prandtl number respectively.

After dropping out the “•” notation Eqs. (2–5) reduced to

7 $$\frac{\partial v(r,t)}{\partial t}=\left(1+\alpha \frac{\partial }{\partial t}\right)\left(\frac{{\partial }^{2}}{\partial {r}^{2}}+\frac{1}{r}\frac{\partial }{\partial r}\right)v(r,t)+Gr\theta (T-{T}_{\infty }),\,r\in (0,1),\,t > 0,$$

8 $$\frac{\partial \theta (r,t)}{\partial t}={\rm{\Pr }}\left(\frac{{\partial }^{2}}{\partial {r}^{2}}+\frac{1}{r}\frac{\partial }{\partial r}\right)\theta (r,t),r\in (0,1),t > 0,$$

9 $$v(r,0)=0,\theta (r,0)=0,\,r\in [0,1],$$

10 $$v(1,t)=H(t)\exp (i\omega t),\theta (1,t)=1,\,t > 0.$$

### Temperature field

Taking the Laplace transform^36^ of Eqs. (8), (10) and keeping in mind Eq. (9),

11 $$q\overline{\theta }(r,q)=\frac{1}{{\rm{\Pr }}}\left(\frac{{\partial }^{2}}{\partial {r}^{2}}+\frac{1}{r}\frac{\partial }{\partial r}\right)\overline{\theta }(r,q),$$

12 $$\overline{\theta }(1,q)=\frac{1}{q}.$$

Now applying finite Hankel transform^36^ of order zero on Eq. (11)

13 $${\overline{\theta }}_{H}(r,q)=\frac{{J}_{1}({r}_{n})}{{r}_{n}}\left(\frac{1}{q}-\frac{1}{q+{{r}_{n}}^{2}/{\rm{\Pr }}}\right),$$

with its inverse Laplace transform

14 $${\theta }_{H}(r,q)=\frac{{J}_{1}({r}_{n})}{{r}_{n}}-\frac{{J}_{1}({r}_{n})}{{r}_{n}}\exp (-({{r}_{n}}^{2}/{\rm{\Pr }})t),$$

where, *r*_*n*_ are positive roots of Bessel function.

Finally, apply inverse Hankel transform we get

15 $$\theta (r,t)=1-2\mathop{\sum }\limits_{n=1}^{\infty }\,\frac{{J}_{0}(r{r}_{n})}{{r}_{n}{J}_{1}({r}_{n})}\exp (-(\,{{r}_{n}}^{2}/{\rm{\Pr }})t),$$

the temperature distribution for the fluid.

Temperature gradient at the surface is equal to dimensionless number Nusselt number and it provides a measure of the convection heat transfer occurring at the surface^37^. So, in order to study the heat transfer from the cylinder surface to the fluid we determine the Nusselt number.

16 $$Nu=-{\left(\frac{\partial \theta (r,t)}{\partial r}\right)}_{r=1}=2\mathop{\sum }\limits_{n=1}^{\infty }\,\exp (-({{r}_{n}}^{2}/{\rm{\Pr }})t).$$

### Velocity field

Applying the Laplace transform to Eqs. (7), (10) and using Eq. (9), the transformed problem is

17 $$q\bar{v}(r,q)=(1+\alpha q)\left(\frac{{\partial }^{2}}{\partial {r}^{2}}+\frac{1}{r}\frac{\partial }{\partial r}\right)\bar{v}(r,q)+Gr\bar{\theta }(r,q),$$

$$\bar{v}(1,q)=\frac{1}{q-i\omega }.$$

Applying the finite Hankel transform to Eq. (17)^36^

18 $$\begin{array}{c}{\bar{v}}_{H}({r}_{n},q)=(1+\alpha q)\left[\frac{1}{(q-i\omega )(q+{{r}_{n}}^{2})}{r}_{n}{J}_{1}({r}_{n})\right]+Gr\frac{{J}_{1}({r}_{n})}{{r}_{n}}\\ \,\left[\frac{1}{q(q+{{r}_{n}}^{2}}-\frac{1}{(q+{{r}_{n}}^{2})(q+\,{{r}_{n}}^{2}/{\rm{\Pr }})}\right].\end{array}$$

Applying inverse Laplace transform to above equation^36^, we obtain

19 $$\begin{array}{rcl}{v}_{H}({r}_{n},q) & = & \frac{{J}_{1}({r}_{n})}{{r}_{n}}\exp (i\omega t)-\frac{{J}_{1}({r}_{n})}{{r}_{n}}\frac{{\omega }^{2}}{{{r}_{n}}^{4}+{\omega }^{2}}\exp (i\omega t)-\frac{{J}_{1}({r}_{n})}{{r}_{n}}\frac{i\omega {{r}_{n}}^{2}}{{{r}_{n}}^{4}+{\omega }^{2}}\exp (i\omega t)\\  &  & -\,\frac{{{r}_{n}}^{3}{J}_{1}({r}_{n})}{{{r}_{n}}^{4}+{\omega }^{2}}\exp (-{{r}_{n}}^{2}t)+i\frac{\omega {r}_{n}{J}_{1}({r}_{n})}{{{r}_{n}}^{4}+{\omega }^{2}}\exp (-{{r}_{n}}^{2}t)+\alpha [\frac{i\omega {J}_{1}({r}_{n})}{{r}_{n}}\exp (i\omega t)\\  &  & -i\,\frac{{J}_{1}({r}_{n})}{{r}_{n}}\frac{{\omega }^{2}(\omega -i{{r}_{n}}^{2})}{{{r}_{n}}^{4}+{\omega }^{2}}\exp (i\omega t)-\frac{{{r}_{n}}^{5}{J}_{1}({r}_{n})}{{{r}_{n}}^{4}+{\omega }^{2}}\exp (\,-\,{{r}_{n}}^{2}t)+i\frac{\omega {{r}_{n}}^{3}{J}_{1}({r}_{n})}{{{r}_{n}}^{4}+{\omega }^{2}}\exp (-{{r}_{n}}^{2}t)]\\  &  & +\frac{Gr{J}_{1}({r}_{n})}{{{r}_{n}}^{3}}+\frac{Gr{J}_{1}({r}_{n})}{{{r}_{n}}^{3}({\rm{\Pr }}-1)}\exp (\,-\,{{r}_{n}}^{2}t)+\frac{Gr{\rm{\Pr }}{J}_{1}({r}_{n})}{{{r}_{n}}^{3}({\rm{\Pr }}-1)}\exp \left(\frac{-{{r}_{n}}^{2}t}{{\rm{\Pr }}}\right);{\rm{\Pr }}\ne 1.\end{array}$$

Finally, applying the inverse Hankel transform^36^ the expression for the velocity field is obtained

20 $$\begin{array}{rcl}v(r,t) & = & \exp (i\omega t)-2{\omega }^{2}\mathop{\sum }\limits_{n=1}^{\infty }\,\frac{{J}_{0}(r{r}_{n})}{{r}_{n}({{r}_{n}}^{4}+{\omega }^{2}){J}_{1}({r}_{n})}\exp (i\omega t)-2i\omega \mathop{\sum }\limits_{n=1}^{\infty }\,\frac{{r}_{n}{J}_{0}(r{r}_{n})}{({{r}_{n}}^{4}+{\omega }^{2}){J}_{1}({r}_{n})}\,\exp (i\omega t)\\  &  & -2\mathop{\sum }\limits_{n=1}^{\infty }\,\frac{{{r}_{n}}^{3}{J}_{0}(r{r}_{n})}{({{r}_{n}}^{4}+{\omega }^{2}){J}_{1}({r}_{n})}\exp (-{{r}_{n}}^{2}t)+2i\omega \mathop{\sum }\limits_{n=1}^{\infty }\,\frac{{r}_{n}{J}_{0}(r{r}_{n})}{({{r}_{n}}^{4}+{\omega }^{2}){J}_{1}({r}_{n})}\exp (-{{r}_{n}}^{2}t)\\  &  & +\alpha [i\omega \exp (i\omega t)-i2{\omega }^{3}\mathop{\sum }\limits_{n=1}^{\infty }\,\frac{{J}_{0}(r{r}_{n})}{{r}_{n}({{r}_{n}}^{4}+{\omega }^{2}){J}_{1}({r}_{n})}\exp (i\omega t)-2{\omega }^{2}\mathop{\sum }\limits_{n=1}^{\infty }\,\frac{{r}_{n}{J}_{0}(r{r}_{n})}{({{r}_{n}}^{4}+{\omega }^{2}){J}_{1}({r}_{n})}\exp (i\omega t)\\  &  & -2\mathop{\sum }\limits_{n=1}^{\infty }\,\frac{{{r}_{n}}^{5}{J}_{0}(r{r}_{n})}{({{r}_{n}}^{4}+{\omega }^{2}){J}_{1}({r}_{n})}\exp (-{{r}_{n}}^{2}t)+i2\omega \mathop{\sum }\limits_{n=1}^{\infty }\,\frac{{{r}_{n}}^{3}{J}_{0}(r{r}_{n})}{({{r}_{n}}^{4}+{\omega }^{2}){J}_{1}({r}_{n})}\exp (-{{r}_{n}}^{2}t)]\\  &  & +\frac{2Gr}{\Pr -1}\mathop{\sum }\limits_{n=1}^{\infty }\,\left[[({\rm{\Pr }}-1)+\exp (-{{r}_{n}}^{2}t)-{\rm{\Pr }}\,\exp \left(\frac{-{{r}_{n}}^{2}t}{{\rm{\Pr }}}\right)]\frac{{J}_{0}(r{r}_{n})}{{r}_{n}{J}_{1}({r}_{n})}\right];{\rm{\Pr }}\ne 1.\end{array}$$

#### Cosine oscillation

The velocity field corresponding to cosine oscillation can be obtained form Eq. (20) by taking its real part. Further this solution can be separated into two parts transient and post-transient solution.

$${v}_{c}(r,t)={v}_{cp}(r,t)+{v}_{ct}(r,t).$$

$$\begin{array}{ll}{v}_{cp}(r,t)= & \cos \,\omega t-2{\omega }^{2}\mathop{\sum }\limits_{n=1}^{\infty }\,\frac{{J}_{0}(r{r}_{n})}{{r}_{n}({{r}_{n}}^{4}+{\omega }^{2}){J}_{1}({r}_{n})}\,\cos \,\omega t-2\omega \mathop{\sum }\limits_{n=1}^{\infty }\,\frac{{r}_{n}{J}_{0}(r{r}_{n})}{({{r}_{n}}^{4}+{\omega }^{2}){J}_{1}({r}_{n})}\,\sin \,\omega t\\  & +\,\alpha [\omega \,\sin \,\omega t-2{\omega }^{3}\mathop{\sum }\limits_{n=1}^{\infty }\,\frac{{J}_{0}(r{r}_{n})}{{r}_{n}({{r}_{n}}^{4}+{\omega }^{2}){J}_{1}({r}_{n})}\,\sin \,\omega t\\  & -2{\omega }^{2}\mathop{\sum }\limits_{n=1}^{\infty }\,\frac{{r}_{n}{J}_{0}(r{r}_{n})}{({{r}_{n}}^{4}+{\omega }^{2}){J}_{1}({r}_{n})}\,\cos \,\omega t]+2Gr\mathop{\sum }\limits_{n=1}^{\infty }\,\frac{{J}_{0}(r{r}_{n})}{{{r}_{n}}^{2}{J}_{1}({r}_{n})},\end{array}$$

$$\begin{array}{rcl}{v}_{ct}(r,t) & = & -2\mathop{\sum }\limits_{n=1}^{\infty }\,\frac{{{r}_{n}}^{3}{J}_{0}(r{r}_{n})}{({{r}_{n}}^{4}+{\omega }^{2}){J}_{1}({r}_{n})}\exp (\,-{{r}_{n}}^{2}t)-2\alpha \mathop{\sum }\limits_{n=1}^{\infty }\,\frac{{{r}_{n}}^{5}{J}_{0}(r{r}_{n})}{({{r}_{n}}^{4}+{\omega }^{2}){J}_{1}({r}_{n})}\exp (\,-\,{{r}_{n}}^{2}t)\\  &  & +\,\frac{2Gr}{{\rm{\Pr }}-1}\mathop{\sum }\limits_{n=1}^{\infty }\,\frac{{J}_{0}(r{r}_{n})}{{{r}_{n}}^{3}{J}_{1}({r}_{n})}\left(\exp (\,-\,{{r}_{n}}^{2}t)-{\rm{\Pr }}\,\exp \left(\frac{-{{r}_{n}}^{2}t}{\Pr }\right)\right);{\rm{\Pr }}\ne 1.\end{array}$$

#### Sine oscillation

Similarly, the velocity field corresponding to sine oscillation can be obtained form Eq. (20) by taking its imaginary part. Further this solution can be separated into two parts transient and post-transient solution

$${v}_{s}(r,t)={v}_{sp}(r,t)+{v}_{st}(r,t),$$

$$\begin{array}{rcl}{v}_{sp}(r,t) & = & \sin \,\omega t-2{\omega }^{2}\mathop{\sum }\limits_{n\mathrm{=1}}^{\infty }\,\frac{{J}_{0}(r{r}_{n})}{{r}_{n}({{r}_{n}}^{4}+{\omega }^{2}){J}_{1}({r}_{n})}\,\sin \,\omega t-2\omega \mathop{\sum }\limits_{n\mathrm{=1}}^{\infty }\,\frac{{r}_{n}{J}_{0}(r{r}_{n})}{({{r}_{n}}^{4}+{\omega }^{2}){J}_{1}({r}_{n})}\,\cos \,\omega t\\  &  & +\alpha [\omega \,\sin \,\omega t-2{\omega }^{3}\mathop{\sum }\limits_{n\mathrm{=1}}^{\infty }\,\frac{{J}_{0}(r{r}_{n})}{{r}_{n}({{r}_{n}}^{4}+{\omega }^{2}){J}_{1}({r}_{n})}\,\cos \,\omega t-2{\omega }^{2}\mathop{\sum }\limits_{n\mathrm{=1}}^{\infty }\,\frac{{r}_{n}{J}_{0}(r{r}_{n})}{({{r}_{n}}^{4}+{\omega }^{2}){J}_{1}({r}_{n})}\,\sin \,\omega t],\end{array}$$

$${v}_{st}(r,t)=2\omega \mathop{\sum }\limits_{n=1}^{\infty }\,\frac{{r}_{n}{J}_{0}(r{r}_{n})}{({{r}_{n}}^{4}+{\omega }^{2}){J}_{1}({r}_{n})}\exp (-{{r}_{n}}^{2}t)+\alpha 2\omega \mathop{\sum }\limits_{n=1}^{\infty }\,\frac{{{r}_{n}}^{3}{J}_{0}(r{r}_{n})}{({{r}_{n}}^{4}+{\omega }^{2}){J}_{1}({r}_{n})}\exp (-{{r}_{n}}^{2}t).$$

### Special case

By taking *α* → 0 in Eq. (20) velocity corresponding to Newtonian fluid is recover already obtained by Khan *et al*.^35^.

21 $$\begin{array}{lll}v(r,t) & = & \exp (i\omega t)-2{\omega }^{2}\mathop{\sum }\limits_{n=1}^{\infty }\,\frac{{J}_{0}(r{r}_{n})}{{r}_{n}({{r}_{n}}^{4}+{\omega }^{2}){J}_{1}({r}_{n})}\exp (i\omega t)-2i\omega \mathop{\sum }\limits_{n=1}^{\infty }\,\frac{{r}_{n}{J}_{0}(r{r}_{n})}{({{r}_{n}}^{4}+{\omega }^{2}){J}_{1}({r}_{n})}\exp (i\omega t)\\  &  & -2\mathop{\sum }\limits_{n=1}^{\infty }\,\frac{{{r}_{n}}^{3}{J}_{0}(r{r}_{n})}{({{r}_{n}}^{4}+{\omega }^{2}){J}_{1}({r}_{n})}\exp (-{{r}_{n}}^{2}t)+2i\omega \mathop{\sum }\limits_{n=1}^{\infty }\,\frac{{r}_{n}{J}_{0}(r{r}_{n})}{({{r}_{n}}^{4}+{\omega }^{2}){J}_{1}({r}_{n})}\exp (-{{r}_{n}}^{2}t)\\  &  & +\frac{2Gr}{\Pr -1}\mathop{\sum }\limits_{n=1}^{\infty }\,\left[[({\rm{\Pr }}-1)+\exp (-{{r}_{n}}^{2}t)-{\rm{\Pr }}\,\exp \left(\frac{-{{r}_{n}}^{2}t}{{\rm{\Pr }}}\right)]\frac{{J}_{0}(r{r}_{n})}{{r}_{n}{J}_{1}({r}_{n})}\right];{\rm{\Pr }}\ne 1.\end{array}$$

#### Cosine oscillation

The velocity field corresponding to Newtonian fluid for cosine oscillation can be obtained form Eq. (21) by taking its real part. Further this solution can be separated into two parts transient and post-transient solution.

$$\begin{array}{rcl}{v}_{cp}(r,t) & = & \cos \,\omega t-2{\omega }^{2}\mathop{\sum }\limits_{n=1}^{\infty }\,\frac{{J}_{0}(r{r}_{n})}{{r}_{n}({{r}_{n}}^{4}+{\omega }^{2}){J}_{1}({r}_{n})}\,\cos \,\omega t\\  &  & -2\omega \mathop{\sum }\limits_{n=1}^{\infty }\,\frac{{r}_{n}{J}_{0}(r{r}_{n})}{({{r}_{n}}^{4}+{\omega }^{2}){J}_{1}({r}_{n})}\,\sin \,\omega t+2Gr\mathop{\sum }\limits_{n=1}^{\infty }\,\frac{{J}_{0}(r{r}_{n})}{{{r}_{n}}^{2}{J}_{1}({r}_{n})}.\end{array}$$

$$\begin{array}{rcl}{v}_{ct}(r,t) & = & -2\mathop{\sum }\limits_{n=1}^{\infty }\,\frac{{{r}_{n}}^{3}{J}_{0}(r{r}_{n})}{({{r}_{n}}^{4}+{\omega }^{2}){J}_{1}({r}_{n})}\exp (\,-{{r}_{n}}^{2}t)+\frac{2Gr}{{\rm{\Pr }}-1}\mathop{\sum }\limits_{n=1}^{\infty }\,\frac{{J}_{0}(r{r}_{n})}{{{r}_{n}}^{3}{J}_{1}({r}_{n})}\\  &  & \times \,\left(\exp (-{{r}_{n}}^{2}t)-{\rm{\Pr }}\,\exp \left(\frac{-{{r}_{n}}^{2}t}{{\rm{\Pr }}}\right)\right).\end{array}$$

#### Sine oscillation

Similarly, the velocity field corresponding to Newtonian fluid for sine oscillation can be obtained form Eq. (21) by taking its imaginary part. Further this solution can be separated into two parts transient and post-transient solution.

$$\begin{array}{l}{v}_{sp}(r,t)=\,\sin \,\omega t-2{\omega }^{2}\mathop{\sum }\limits_{n=1}^{\infty }\,\frac{{J}_{0}(r{r}_{n})}{{r}_{n}({{r}_{n}}^{4}+{\omega }^{2}){J}_{1}({r}_{n})}\,\sin \,\omega t-2\omega \mathop{\sum }\limits_{n=1}^{\infty }\,\frac{{r}_{n}{J}_{0}(r{r}_{n})}{({{r}_{n}}^{4}+{\omega }^{2}){J}_{1}({r}_{n})}\,\cos \,\omega t.\end{array}$$

$${v}_{st}(r,t)=2\omega \mathop{\sum }\limits_{n=1}^{\infty }\,\frac{{r}_{n}{J}_{0}(r{r}_{n})}{({{r}_{n}}^{4}+{\omega }^{2}){J}_{1}({r}_{n})}\exp (-{{r}_{n}}^{2}t).$$

## Numerical Results

**Figure 2:**
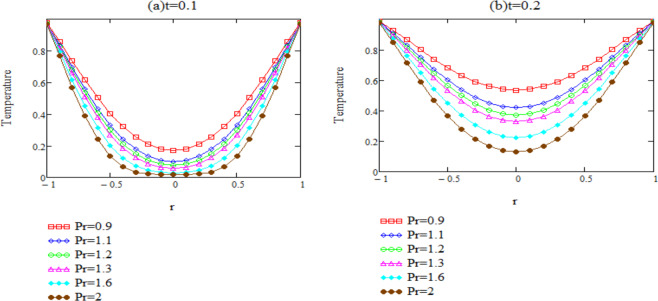
*θ*(*r*, *t*) corresponding to different values of Pr.

**Figure 3:**
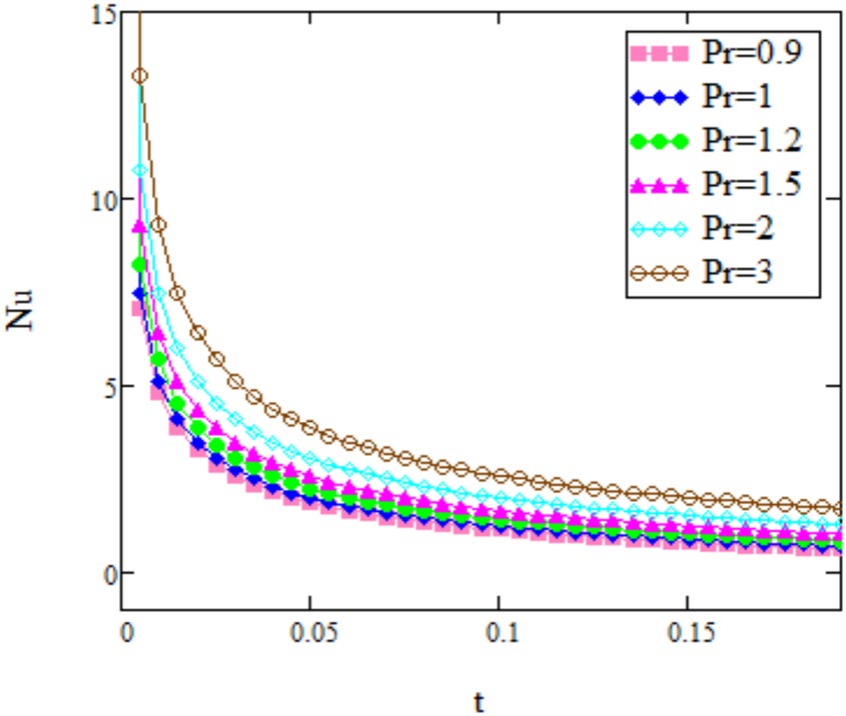
*Nu* corresponding to different values of Pr.

**Figure 4:**
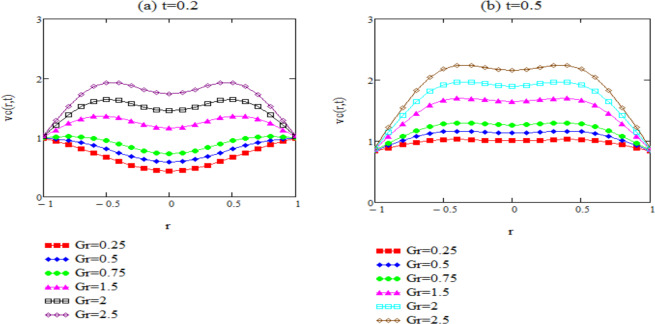
*v*_*ct*_(*r*, *t*) corresponding to different values of Gr.

**Figure 5:**
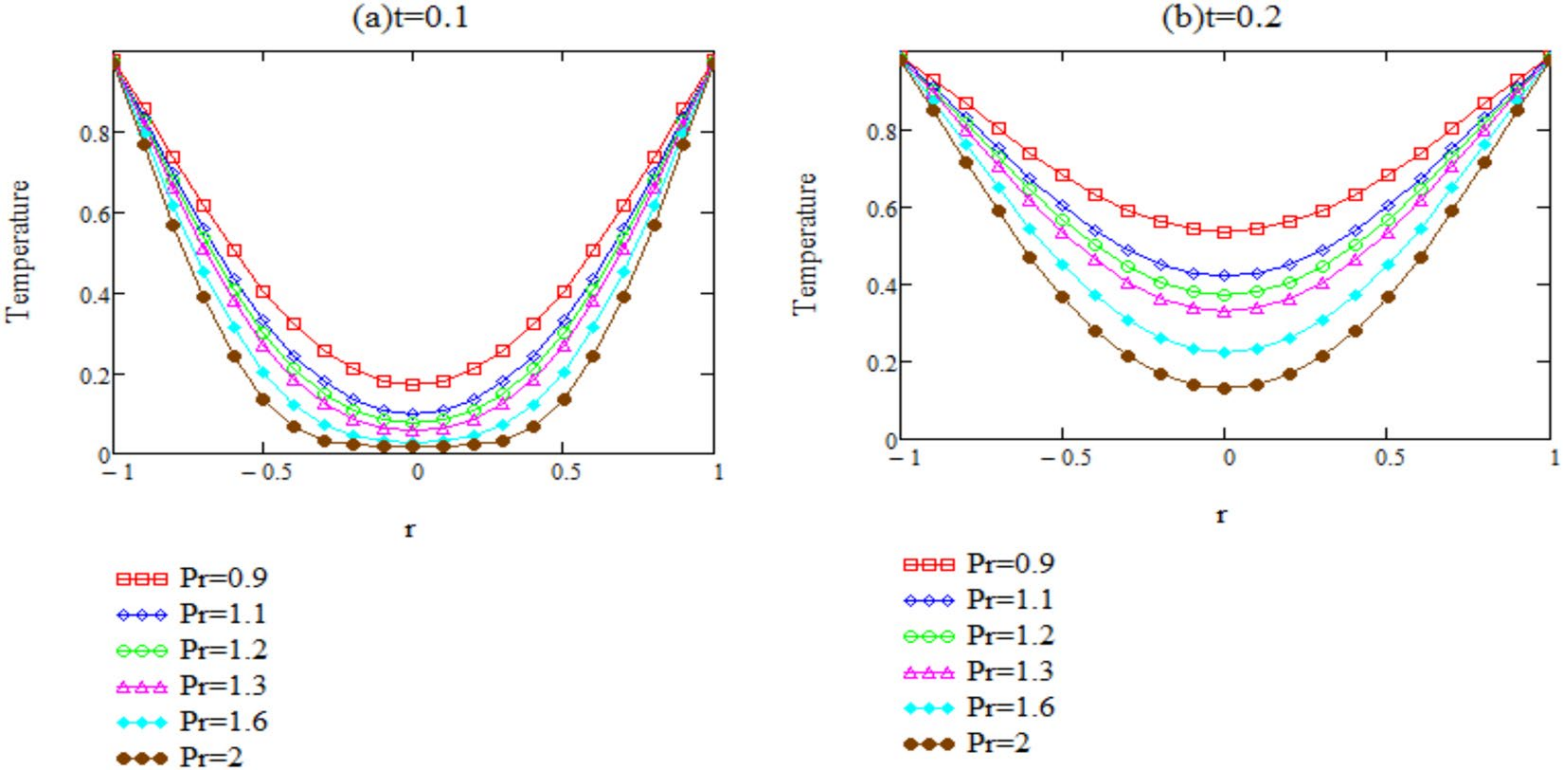
*v*_*ct*_(*r*, *t*) corresponding to Different values of Pr.

**Figure 6:**
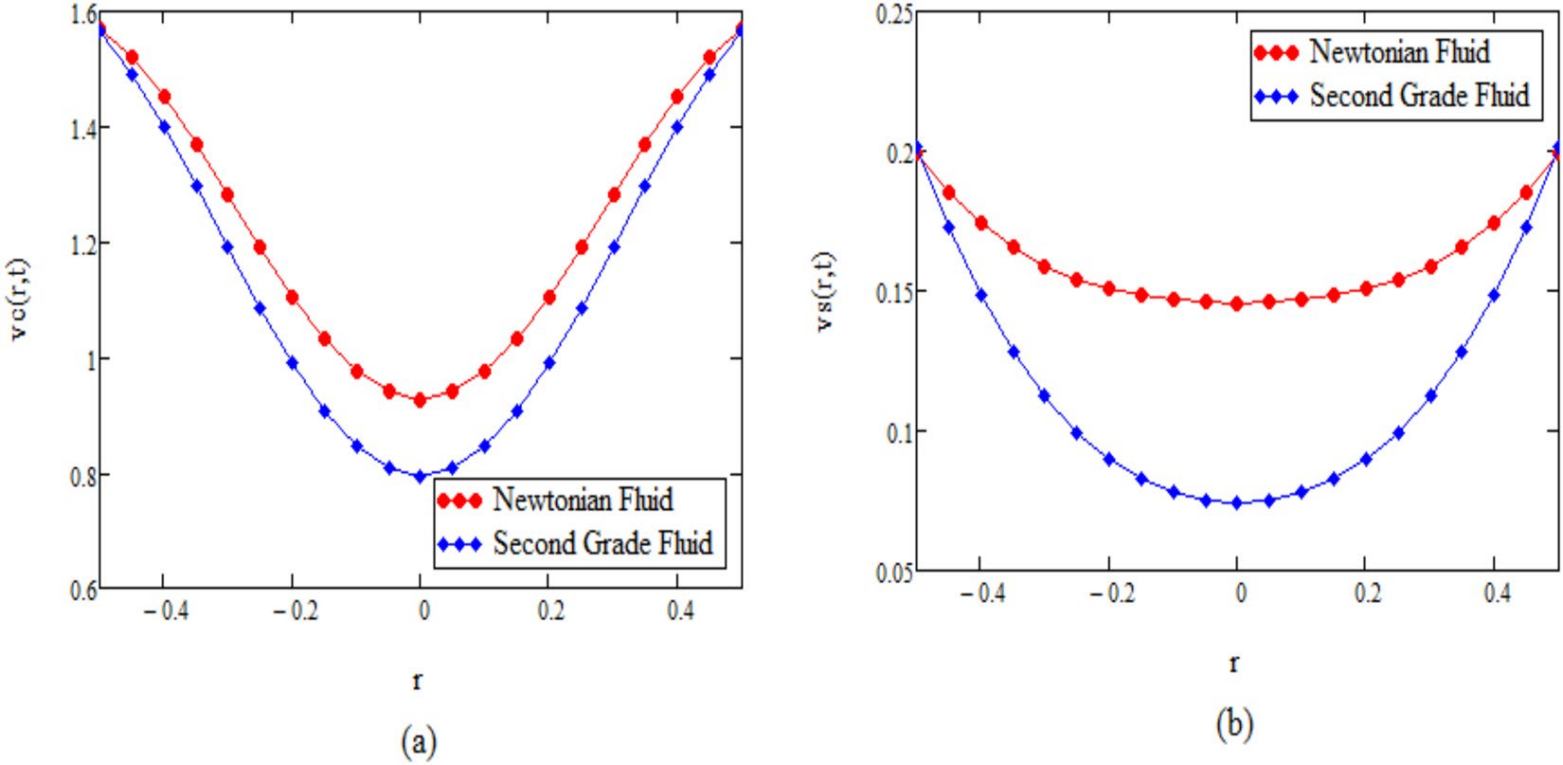
(**a**) Comparison for Newtonian and second grade fluid for *v*_*ct*_(*r*, *t*), (**b**) Comparison for Newtonian and second grade fluid for *v*_*st*_(*r*, *t*).

Unsteady natural convection flow of second grade fluid through an oscillating infinite vertical cylinder is investigated in this study. The dimensionless governing equations for temperature and velocity are obtained by introducing the non-dimensional variables, Their exact solutions are computed by means of integral transformation. Some numerical results have been presented for different parameters in the form of graphs and depicted in Figs. (2–6) by using MATHCAD.

Figure 2 is plotted with a specific goal to examine the impact of the *Pr* on the fluid temperature. For time *t* and *Pr*, curves relating to the dimensionless temperature are sketched versus the radial coordinate. Graphically it is pertinent that as the values of Prandtl number *Pr* enhances, fluid’s temperature decreases. As expected, increasing *Pr* reduces the thermal conductivity and enhances the viscousness of the fluid which results in reduction of thermal boundary layer thickness. From Eq. (15) we can observe that the exponential term tends fast to zero as time increases or, value of Prandtl number decreases. The heat transfer between the fluid and boundary is significant. From the central part of the graph we can say that fluid is not heated for small values of time and it heated as time increases.

Figure 3 represents the effect of *Nu* for variation in *Pr*. The Nusselt number is increases by increase in *Pr*. For small *t*, *Nu* is greater which implies that for smaller *t* the convection is extremely productive. For expansive estimations of the time *t*, the conduction is overwhelming and the heat exchange is created just by conduction for huge estimations of the time *t* (for *t* → 1, *Nu* → 0). With the decrease in Prandtl number the area of thermal boundary layer increases, therefore, the temperature gradient decreases with *Pr*.

Figure 4 is to check the effect of Grashof number on cosine velocity function. We take *Pr* = 0.75, *ω* = 1.5. It must be accentuated that, for smaller values of *Gr* the fluid velocity has low values. This is due the effect of the temperature on the fluid velocity solution. Low values of the *Gr* lead to reduced contribution of the temperature in the fluid flow, therefore, the viscous forces increases and the velocity decreases and vice versa for larger values of *Gr*.

Figure 5 depicted for velocity cosine function for variation in *Pr*. We take *Gr* = 0.7, *ω* = 1.7. For larger *Pr*, fluid velocity decreases. The buoyancy forces created by the density differences are high for the smaller values of the Prandtl number when the temperature is high. When the Prandtl number is large, the viscous damping action becomes bigger and fluid velocity decreases.

The decrease in the transient solution *v*_*ct*_(*r*, *t*) and *v*_*st*_(*r*, *t*) can be observe from the Tables 1 and 2 respectively. From Table 1 clearly we can observe that by increasing in time up to *t* = 31 solution for *v*_*ct*_(*r*, *t*) approaches to zero. From Table 2 clearly we can observe that by increasing in time up to *t* = 6 solution for *v*_*st*_(*r*, *t*) approaches to zero.

**Table 1:** Diminishing of *v*_*ct*_(*r*, *t*), for Gr = 5, Pr = 5 and *ω* = 0.449.

r	*v*_*ct*_(*r*, 0.5)	*v*_*ct*_(*r*, 5)	*v*_*ct*_(*r*, 31)
−0.5	−0.948827	−0.004779	−1.39 × 10^−15^
−0.4	−1.095831	−0.005536	−1.61 × 10^−15^
−0.3	−1.215995	−0.006159	−1.79 × 10^−15^
−0.2	−1.304947	−0.006624	−1.92 × 10^−15^
−0.1	−1.359563	−0.006911	−2 × 10^−15^
0	−1.377975	−0.007008	−2.03 × 10^−15^
0.1	−1.359563	−0.006911	−2 × 10^−15^
0.2	−1.304947	−0.006624	−1.92 × 10^−15^
0.3	−1.215995	−0.006159	−1.79 × 10^−15^
0.4	−1.095831	−0.005536	−1.61 × 10^−15^
0.5	−0.948827	−0.004779	−1.39 × 10^−15^

**Table 2:** Diminishing of *v*_*st*_(*r*, *t*), for *ω* = 0.9.

r	*v*_*st*_(*r*, 0.1)	*v*_*st*_(*r*, 0.5)	*v*_*st*_(*r*, 6)
−0.5	−6.039034	−0.646567	−3.55 × 10^−14^
−0.4	−6.841784	−0.748936	−4.11 × 10^−14^
−0.3	−7.458591	−0.833287	−4.57 × 10^−14^
−0.2	−7.89051	−0.896156	−4.92 × 10^−14^
−0.1	−8.144732	−0.93495	−5.13 × 10^−14^
0	−8.228494	−0.948063	−5.2 × 10^−14^
0.1	−8.144732	−0.93495	−5.13 × 10^−14^
0.2	−7.89051	−0.896156	−4.92 × 10^−14^
0.3	−7.458591	−0.833287	−4.57 × 10^−14^
0.4	−6.841784	−0.748936	−4.11 × 10^−14^
0.5	−6.039034	−0.646567	−3.55 × 10^−14^

Jamil *et al*.^10^ obtained exact solutions for the motion of a fractionalized second grade fluid, by applying limits on fractional parameter we reduce the result to second grade fluid and compare with our solution Table 3. Fetecau *et al*.^11^ obtained the solutions for the oscillating motion of a generalized Burgers fluid due to longitudinal oscillations of an infinite circular cylinder. By applying some limits we reduce the solutions for second grade fluid and compare with our calculated results Table 4.

**Table 3:** Comparison of current result with the results calculated by Jamil *et al*.^10^.

r	*v*_*st*_(*r*, *t*) Current Solution	*w*_*st*_(*r*, *t*) Jamil *et al*. [10]	*w*_*st*_(*r*, *t*) − *v*_*st*_(*r*, *t*)
0.1	0.1697	0.1558	−0.013
0.12	0.1689	0.1789	0.009
0.14	0.168	0.1979	0.029
0.16	0.1669	0.2124	0.045
0.18	0.1656	0.2222	0.056
0.2	0.1641	0.2270	0.062
0.22	0.1625	0.2268	0.064
0.24	0.1607	0.2217	0.061
0.26	0.1586	0.2118	0.053
0.28	0.1564	0.1975	0.041
0.3	0.1539	0.1791	0.025
0.32	0.1512	0.1572	0.005
0.34	0.1482	0.1323	−0.015
0.36	0.145	0.1051	−0.039
0.38	0.1416	0.0764	−0.065
0.4	0.1378	0.04681	−0.091

**Table 4:** Comparison of current result with the results calculated by Fetecau *et al*.^11^.

r	*v*_*st*_(*r*, *t*) Current Solution	*w*_*st*_(*r*, *t*) Fetecau *et al*.^11^	*w*_*st*_(*r*, *t*) − *v*_*st*_(*r*, *t*)
0	1.3858	1.3809	−0.005
0.01	1.3859	1.3828	−0.003
0.02	1.386	1.386	0
0.03	1.3862	1.3884	0.002
0.04	1.3864	1.3914	0.005
0.05	1.3866	1.3940	0.007
0.06	1.3870	1.3959	0.008
0.07	1.3874	1.3966	0.009
0.08	1.3878	1.3957	0.007
0.09	1.3883	1.3929	0.004
0.1	1.3889	1.3878	−0.001

In Fig. 6 and Table 5 the velocities of the Newtonian and second grade fluids compared at same values of the parameters. From this figure we observe that velocity of the Newtonian fluid is greater in comparison to second grade fluid. This validate our results as Newtonian fluid offer low resistance to fluid flow as compared to second grade fluid.

**Table 5:** Comparison for Newtonian and second grade fluid.

r	*v*_*st*_(*r*, *t*) Newtonian Fluid Khan *et al*.^35^	*v*_*st*_(*r*, *t*) Second Grade Fluid	*v*_*ct*_(*r*, *t*) Newtonian Fluid Khan *et al*.^35^	*v*_*ct*_(*r*, *t*) Second Grade Fluid
0	0.506643264	0.463544613	0.052821001	0.043338735
0.05	0.519850786	0.476558765	0.052876382	0.043392608
0.1	0.558687336	0.514817095	0.053048011	0.043559719
0.15	0.62084455	0.576016892	0.053351903	0.043856089
0.2	0.702637486	0.656482586	0.053813303	0.044306983
0.25	0.799236894	0.751397948	0.054464625	0.044944843
0.3	0.904972724	0.855109495	0.055342757	0.045806603
0.35	1.01368897	0.961481193	0.056485937	0.04693057
0.4	1.119126643	1.064277262	0.05793039	0.048353064
0.45	1.215309935	1.157548133	0.059706981	0.050105078
0.5	1.296910568	1.235994547	0.061838094	0.052209162

## Conclusion

Unsteady natural convection flow of second grade over an oscillating infinite vertical cylinder is investigated. The exact solutions for temperature and velocity fields are computed by means of integral transformations. Following important points are observed from the studied problem

- Temperature is an increasing function of *Pr*.
- The Nusselt number is increasing function of *Pr*.
- Fluid velocity increasing with respect to *Gr* and decreases for *Pr*.
- The transient solutions are noteworthy up to 10^−14^, after that point the fluid moves according with the post-transient solution.
- The velocity of the Newtonian fluid is greater in comparison to second grade fluid.
- This validate our results as Newtonian fluid offer low resistance to fluid flow as compared to second grade fluid.
